# Correlation analysis of the combined detection of serum CEA, CA72-4, CA19-9 and PGI and postoperative recurrence of gastric cancer

**DOI:** 10.5937/jomb0-59430

**Published:** 2026-02-27

**Authors:** Bing Han, Yuli Yan, Yanyan Zhong, Xiaoou Li, Yong Zhang

**Affiliations:** 1 Chongqing Red Cross Hospital (People's Hospital of Jiangbei District), Department of Gastroenterology, Chongqing, China; 2 Fujian Medical University First Affiliated Hospital, Department of Gastroenterology, Fuzhou, China; 3 Jiangxi Province Hospital of Integrated Chinese and Western Medicine, The Second Department of General Surgery, Nanchang, China

**Keywords:** carcinoembryonic antigen (CEA), cancer antigen 72-4 (CA72-4), cancer antigen 19-9 (CA19-9), pepsinogen I (PGI), gastric cancer, karcinoembrionski antigen (CEA), antigen karcinoma 72-4 (CA72-4), antigen karcinoma 19-9 (CA19-9), pepsinogen I (PGI), karcinom želuca

## Abstract

**Background:**

To determine if serum CEA, CA72-4, CA19-9, and PGI in gastric cancer patients following radical gastrectomy are associated with postoperative recurrence.

**Methods:**

The gastric cancer group consisted of 102 patients who were admitted to our hospital between January 2022 and June 2024 and had undergone radical gastrectomy (RG); the control group consisted of 34 healthy volunteers who were examined during the same period. Based on whether there was a recurrence following the procedure, patients with gastric cancer were split into two groups: 87 patients who did not experience a recurrence and 15 patients who did. The control group's serum levels of CEA, CA72-4, CA19-9, and PGI were assessed during physical examination, the day before surgery for gastric cancer patients, and at follow-up (or recurrence) one year later.

**Results:**

In the gastric cancer group, serum levels of PGI were lower than in the control group, whereas CEA, CA72-4, and CA19-9 were greater (all P&lt; 0.05). The recurrence rate (15/102 patients) was 14.71%. Poor differentiation degree, decreased PGI, and TNM stage III disease were all independent risk variables for recurrence following RG.

**Conclusions:**

Patients with RG who had elevated CEA, CA72-4, and CA19-9, decreased PGI, TNM stage III, and little differentiation are at independent risk for postoperative recurrence.

## Abstract (sr)

**Uvod:**

Cilj ovog istraživanja je bio da se ispita povezanost serumskih nivoa karcinoembrionskog antigena (CEA), antigena karcinoma 72-4 (CA72-4), antigena karcinoma 19-9 (CA19-9) i pepsinogena I (PGI) sa postoperativnim recidivom kod pacijenata sa karcinomom želuca nakon radikalne gastrektomije.

**Metode:**

U studiju je uključeno 102 pacijenta sa karcinomom želuca, koji su hospitalizovani u našoj ustanovi u periodu od januara 2022. do juna 2024. godine, i kod kojih je sprovedena radikalna gastrektomija (RG). Kontrolnu grupu činila su 34 zdrava dobrovoljca pregledana u istom periodu. Prema prisustvu postoperativnog recidiva, pacijenti sa karcinomom želuca podeljeni su u dve podgrupe: 87 bez recidiva i 15 sa recidivom. Serumske koncentracije CEA, CA72-4, CA19-9 i PGI određivane su kod kontrolne grupe tokom sistematskog pregleda, kod pacijenata sa karcinomom želuca dan pre operacije, te godinu dana nakon zahvata, odnosno u trenutku potvrde recidiva.

**Rezultati:**

Kod pacijenata sa karcinomom želuca zabeleženi su značajno niži nivoi PGI, a viši nivoi CEA, CA72-4 i CA19-9 u poređenju sa kontrolnom grupom (svi P&lt; 0,05).

**Zaključak:**

Povišeni nivoi CEA, CA72-4 i CA19-9, sniženi PGI, TNM stadijum III i nizak stepen histološke diferenciacije predstavljaju nezavisne faktore rizika za postoperativni recidiv kod pacijenata nakon radikalne gastrektomije.

## Introduction

Gastric cancer originates from the mucosal epithelial cells of the stomach, and the most common pathological type is adenocarcinoma [1][2][3][4]. After treatment, such as surgery, chemotherapy or radiotherapy, the five-year survival rate is much lower than that of patients diagnosed with early-stage gastric cancer. Therefore, early judgment of the recurrence situation and its influencing factors after radical resection of gastric cancer (RG) and the formulation of effective treatment plans are the keys to improving the prognosis of patients [5][6][7]. Therefore, the feasibility of using serological indicators in predicting the postoperative recurrence of gastric cancer has always been the focus of clinical research. Relevant studies [8][9][10] have shown that the serum ALB concentration is related to postoperative recurrence, metastasis and survival outcomes after RG. PGI and PG exist mainly in gastric juice and are widely used in the screening of early gastric cancer [11]. Carcinoembryonic antigen (CEA) is a serum marker for the early screening of various cancers and is used mainly to reflect tumour proliferation, migration, and other aspects related to tumour burden [12]. This study retrospectively analysed the relationship between the combined detection of these four indicators and the recurrence rate after RG, aiming to provide objective evidence for predicting recurrence after RG and guiding the clinical selection of reasonable treatment measures.

## Materials and methods

### General information

The gastric cancer group consisted of 102 patients, 61 of whom were male and 41 of whom were female, who were hospitalised to our hospital between January 2022 and June 2024 and who had undergone RG surgery. Age ranged from 46-86 years (68.6±9.02); BMI ranged from 18.32-27.83 kg/m^2^ (23.58±2.43) kg/m^2^. Thirty-four additional healthy volunteers, 20 of whom were male and 14 of whom were female, were examined physically in our hospital over the same period. Age ranged from 33-74 years (56.64±10.58); BMI ranged from 17.93-28.52 kg/m^2^ (21.63±2.42) kg/m^2^.

Exclusion criteria: (1) In-hospital mortality and incomplete clinical data; (2) The presence of malignant tumours or combined with damage to the blood and immune system; (3) Pregnancy or lactation.

Inclusion criteria: (1) Met the diagnostic criteria for gastric cancer in the »International Norms for Diagnosis and Treatment of Gastric Cancer (2022 Edition)«; (2) Met the surgical indications for RG; (3) Initial diagnosis and no previous antitumor treatment; (4) TNM stage I to III.

### Detection of the serum levels of CEA, CA72-4, CA19-9 and PGI

Three millilitres of venous blood was collected from the control group during physical examination, one day before surgery, and one year during follow-up (or at recurrence). Serum CEA and CA19-9 were detected by a Roche e801 fully automatic electrochemiluminescence analyser, and serum CA72-4 was detected by an Antu A2000PLUS fully automatic electrochemiluminescence analyser. A Mindray BS2800 fully automatic biochemical analyser was used to detect serum PGI.

### Clinical data collection

The general data collected included sex, age, smoking status, lymph node metastasis status, lesion differentiation and infiltration, etc;

(2) Peripheral serum indicators, including the levels of CEA, CA72-4, CA19-9, and PGI; (3) Correlations between peripheral serum indicators and recurrence after RG and their predictive value for recurrence.

### Follow-up and grouping

The patients in the gastric cancer group were followed up for one year after the operation via telephone or outpatient reexamination (until July 2024 or until recurrence or death), with follow-up once a month. Gastric cancer patients were divided into a non-recurrence group and a recurrence group based on whether they had a recurrence after the operation. Recurrent patients were diagnosed with gastric cancer of the same pathological type through pathological examination.

### Statistical methods

SPSS 26.0 statistical software was used. Count data are expressed as n (%) for the x^2^ test. The measurement data are expressed as M(P25, P75) and were subjected to t tests or Z tests. Univariate and multivariate logistic regression analyses were conducted to analyse the factors influencing postoperative recurrence in patients with RG. Receiver operating characteristic curves were used to analyse the predictive value of the serum CEA, CA72-4, CA19-9 and PGI for postoperative recurrence in patients with RG. A P value <0.05 was considered to indicate statistical significance.

## Results

### Comparison of the serum CEA, CA72-4, CA19-9 and PGI levels between the gastric cancer group and the control group

The serum PGI level in the gastric cancer group was lower than that in the control group, and the serum CEA, CA72-4 and CA19-9 levels in the gastric cancer group were greater than those in the control group (all P<0.05). See Table 1.

**Table 1:** Comparison of serum CEA, CA72-4, CA19-9 and PG I levels between the gastric cancer group and the control group.

Group	n	CEA (ng/mL)	CA72-4 (U/mL)	CA19-9 (U/mL)	PGI (ng/mL)
Gastric cancer group	102	16.62 (8.49, 24.72)	24.28±8.95	76.86±27.83	49.83±11.32
Control group	34	1.96 (1.13, 2.75)	3.95±0.93	13.54±8.64	89.32±26.54
t/Z value	-	15.914	13.509	8.573	16.958
P value	-	0.004	0.011	0.018	0.001

### Univariate analysis

After follow-up, among the 102 RG patients, 15 experienced postoperative recurrence, with a recurrence rate of 14.71% (15/102 patients). There were 15 patients in the recurrence group and 87 patients in the non-recurrence group. The TNM stage, the degree of tissue differentiation, and the blood levels of CEA, CA72-4, CA19-9, and PGI were all statistically different between the two groups (all P<0.05). See Table 2.

**Table 2:** Univariate analysis of postoperative recurrence in patients with RG.

Group	Recurrence group<br>(n = 15)	Non-recurrence group<br>(n=87)	t/Zlx^2^ value	
Gender (Male/Female)	10/5	51/36	5.191	0.241
Age (Years)	67.86±11.62	68.74±8.57	0.427	0.239
BMI (kg/m^2^)	21.98±1.91	23.37±1.68	2.194	0.735
Smoking history (n)%			4.368	0.327
Yes	8 (53.33)	41 (47.13)		
No	7 (46.67)	46 (52.87)		
History of alcohol consumption (n)%			6.913	0.071
Yes	6 (40.00)	33 (37.93)		
No	9 (60.00)	54 (62.07)		
Tumor location (n)%			0.604	3.897
Lower part of the stomach	4 (26.67)	32 (36.78)		
The middle part of the stomach	3 (20.00)	17 (19.54)		
Upper part of the stomach	8 (53.33)	38 (43.68)		
Tumor size (n)%			6.532	0.159
3 cm	9 (60.00)	43 (49.43)		
<3 cm	6 (40.00)	44 (50.57)		
Tissue differentiation (n)%			15.249	0.001

### Multivariate regression analysis

Multivariate logistic regression analysis based on the results of univariate analysis revealed that elevated CEA, CA72-4, and CA19-9 levels, decreased PGI, TNM stage III, and poorly differentiated degree were all independent risk factors for postoperative recurrence in patients with RG (all P<0.05). See Table 3.

**Table 3:** Multivariate Logistic Regression Analysis of Postoperative Recurrence in patients with RG.

	β	wald (x^2^)	SE	OR	95%CI	P value
Low differentiation	1.154	8.192	0.534	3.236	1.079~8.295	0.033
TNM period	1.027	6.964	0.497	3.299	1.032~8.571	0.039
CEA level	0.078	9.763	0.034	1.124	1.076~8.671	0.008
CA72-4 level	0.571	14.572	0.254	2.585	1.096~4.905	0.005
CA19-9 level	0.071	14.742	0.018	1.138	1.015~1.708	0.004
PGI level	0.029	11.091	0.029	1.029	1.011 ~1.652	0.007

### The predictive value of the serum levels of CEA, CA72-4, CA19-9 and PGI for postoperative recurrence in patients with RG

After analysis, the combined prediction AUC of the serum CEA, CA72-4, CA19-9 and PGI levels for postoperative recurrence in patients with RG was greater than that of the individual predictions. See Table 4.

**Table 4:** Predictive value of serum CEA, CA72-4, CA19-9 and PGI levels for postoperative recurrence in patients with RG.

Factor	AUC	95%CI	Truncation value	Sensitivity	Specificity	Yoden Index
CEA	0.774	0.729~0.818	15.37	85.74	56.83	0.426
CA72-4	0.719	0.628~0.809	50.92	39.46	95.13	0.346
CA19-9	0.792	0.738~0.805	116.97	58.91	78.11	0.370
PGI	0.783	0.724~0.833	65.52	89.46	55.74	0.452
Joint detection	0.894	0.832~0.933	-	85.74	82.79	0.685

## Discussion

The early symptoms of gastric cancer lack specificity and mainly include stomach pain, nausea and vomiting, acid reflux and belching, loss of appetite, etc. [13]. Most patients are initially diagnosed with middle- or advanced-stage gastric cancer. Radical surgery is the most effective method for treating gastric cancer. With the significant progress made in the research of targeted drugs, multidisciplinary treatment based on radical surgery has improved the prognosis of gastric cancer patients after RG [14][15][16]. Owing to the high molecular, biological and histopathological specificity of middle and advanced gastric cancer, the recurrence rate of patients after RG significantly increases, which is not conducive to the prognosis of patients. Predicting the recurrence rate of patients after RG through serum-specific indicators and actively adopting targeted prevention [17].

CEA is an acidic glycoprotein with embryonic antigen characteristics that is located mainly in the cell membrane. The serum CEA level abnormally increases in patients with digestive tract malignancies such as gastric cancer and intestinal cancer. CA72-4 is currently the indicator with the highest correlation with gastric cancer [18][19][2]. It is a carbohydrate macromolecule protein and constitutes the skeleton of tumour cells. It is distributed mainly on the surface of epithelial cells and is superior to other tumour indicators in terms of sensitivity and specificity [20]. CA19-9 is a high molecular weight glycoprotein and is mainly free in serum in the form of a mucin antigen. When digestive tract tumour cells appear, they specifically bind to cell surface receptors. CA72-4 and CA19-9 are expressed at low levels in the serum of normal individuals and are abnormally overexpressed in gastrointestinal tumours [21][22][23].

PGI is a type of pepsin precursor and is mainly distributed in digestive juices to activate pepsin [24].

14.71% of RG patients experienced post-operative recurrence. Multivariate logistic regression ana lysis revealed that elevated CEA, CA72-4, and CA19-9; decreased PGI; TNM stage III; and low differentiation degree were independent risk factors. At stage III of TNM staging, gastric cancer cells break through the mucosal layer, increasing the possibility of lymph node metastasis of the tumour. The AUC of the combined prediction of postoperative recurrence in patients with RG was greater than that of each prediction. The sensitivity of serum CEA was 85.74%, and the specificity was 56.83%. When tumour cells enter the proliferation stage, CEA is overexpressed, and at the same time, CEA is released through the cell membrane into the extracellular tissue fluid and blood [25]. The results of this study are similar, indicating that elevated CEA levels are related to recurrence after RG. The tumour cells of patients with recurrent gastric cancer after RG are considered to have a greater degree of malignancy, and the tumour cells at the lesion site proliferate faster and are more invasive, resulting in a higher serum CEA level [26].

The sensitivity of serum CA72-4 was 39.46%, and the specificity was 95.13%. The increase in serum CA72-4 reflects the accelerated proliferation and division of gastric cancer cells. The residual cancer cells after RG are still in an active proliferating state, and the possibility of recurrence increases. The sensitivity of the serum CA19-9 concentration was 58.91%, and the specificity was 78.11%. Considering that the number of tumour cells in the body was relatively large, many residual microtumor lesions remained after RG. The sensitivity of serum PGI was 89.46%, and the specificity was 55.74%. The level of PGI reflects the degree of atrophy of the gastric mucosal glands and the gastric acid secretion function.

## Conclusion

To sum up, in RG patients, poor differentiation degree, TNM stage III, decreased PGI, increased CEA, CA72-4, and CA19-9 are independent risk factors for postoperative recurrence.

## Dodatak

### Authors' contributions

Bing Han and Yuli Yan contributed equally to this work.

### Conflict of interest statement

All the authors declare that they have no conflict of interest in this work.
